# Comparison of risk factors for Parkinson’s disease, coronary events and ischemic stroke

**DOI:** 10.1038/s41531-022-00374-z

**Published:** 2022-08-25

**Authors:** Lu Song, Shunming Zhang, Huiping Li, Oskar Hansson, Emily Sonestedt, Yan Borné

**Affiliations:** 1 Department of Neurology, Xinhua Hospital, Shanghai Jiaotong University School of Medicine, Shanghai, China; 2 Nutritional Epidemiology Institute and School of Public Health, Tianjin Medical University, Tianjin, China; 3 Department of Clinical Sciences in Malmö, Lund University, Lund, Sweden; 4 Memory Clinic, Skåne University Hospital, Lund, Sweden

**Keywords:** Parkinson's disease, Risk factors

## Abstract

Parkinson’s disease (PD) and cardiovascular disease share many important risk factors, but some associations differ. However, there are no studies that have compared their shared and specific risk factors. The present study aimed to compare risk factors for PD, coronary events, or ischemic stroke. We prospectively analyzed data from 26,210 participants with lifestyle factors aged 45–73 years enrolled between 1991 and 1996. The Cox proportional hazards model was used to calculate hazard ratios (HRs) and 95% confidence intervals (CIs) of PD, coronary events, or ischemic stroke in relation to each factor. A modified Lunn-McNeil competing risk analysis was performed to compare the HR strength of the three outcomes. A total of 486 incident PD cases, 3288 coronary events cases and 2,972 ischemic stroke cases occurred during a mean follow-up of 21 years. In multivariable models, age (per additional year: HR = 1.08; 95% CI: 1.06, 1.09), diabetes (HR = 1.52; 95% CI: 1.02, 2.26), neutrophil–lymphocyte ratio (per SD increase: HR = 1.09; 95% CI: 1.00, 1.19), and fasting blood glucose (per SD increase: HR = 1.18; 95% CI: 1.03, 1.36) are the risk factors for PD, whereas female sex (HR = 0.54; 95% CI: 0.43, 0.67), smoking (current smoker [HR = 0.57; 95% CI: 0.43, 0.74] and former smoker [HR = 0.81; 95% CI: 0.66, 0.99]), HDL (per SD increase: HR = 0.74; 95% CI: 0.57, 0.95), and LDL (per SD increase: HR = 0.77; 95% CI: 0.61, 0.96) are the protective factors. A comparison of risk factors for PD, coronary events, and ischemic stroke showed the three outcomes had concordant and discordant risk factors. Our results indicated the risk factor profiles for PD, coronary events, or ischemic stroke had many similarities, but also significant differences.

## Introduction

Parkinson’s disease (PD) is a slowly progressive neurodegenerative disease affecting ~6 million people worldwide^1^. PD is uncommon before 50 years of age and increases with age thereafter^1^. As life expectancy increases, the prevalence and burden of PD are expected to increase even further^2^. The pathogenic mechanisms of PD remain unknown, and there is currently no cure for PD. Therefore, identifying risk factors of PD is important for the early detection of at-risk subgroups and potential interventions.

The typical risk factors for PD are older age, male sex, diabetes, hypertension, and obesity^3^. PD and cardiovascular disease (CVD) are both prevalent in the elderly population. A recent study reported that PD was associated with a higher risk of developing CVD^4^. Although CVD and PD share many risk factors, some risk factors are discordantly associated with these conditions^3,5^. For example, older age and male sex are each associated with a higher risk of both PD and CVD, whereas smoking and plasma low-density lipoprotein (LDL) cholesterol levels have opposite associations with the two outcomes^3^. Furthermore, from the pathophysiological point of view, inflammation and lipid metabolism may contribute to the development of both PD and CVD^3^. However, no studies have been done to compare the traditional CVD risk factors, inflammation markers, and lipid profiles associated with PD, coronary events, and ischemic stroke. It is important to distinguish the shared and specific risk factors for PD, coronary events, and ischemic stroke, providing information for the clinical strategy.

To date, although several studies have investigated some CVD risk factors for PD, the inclusion of the CVD risk factors was dispersed among different studies and yielded conflicting results^6–9^. Therefore, there were two main aims in this study: the first was to investigate the association of traditional CVD risk factors, inflammatory markers, and lipid profiles with the risk of PD in a prospective population-based cohort; the second was to compare the magnitudes of associations on PD, coronary events, and ischemic stroke, separately.

## Results

### Baseline characteristics

Table 1 shows baseline characteristics of the participants by incident disease status (PD, coronary events, and ischemic stroke). Compare with participants without incident PD, coronary events, or ischemic stroke, participants who developed these outcomes were more likely to be older, be male, consume more total energy, and have hypertension. In addition, compared with participants without incident PD, those with incident PD tended to be never smokers. In contrast, participants who developed coronary events or ischemic stroke were less likely to be never smokers. Furthermore, participants with incident coronary events or ischemic stroke had higher body mass index (BMI), apolipoprotein B (ApoB)/apolipoprotein A1 (ApoA1) ratio, ApoB, fasting blood glucose (FBG), and hemoglobin A1c (HbA1c), but lower educational level, ApoA1, high-density lipoprotein (HDL) cholesterol, and were more likely to have diabetes and take lipid-lowering drugs.

**Table 1:** Baseline characteristics of the study participants by incident disease status (*n* = 26,210)^a^.

Characteristics	Parkinson’s disease		*P*-value^b^	Coronary events		*P*-value^b^	Ischemic stroke		*P*-value^b^
	No (*n* = 25,724)	Yes (*n* = 486)		No (*n* = 22,922)	Yes (*n* = 3288)		No (*n* = 23,238)	Yes (*n* = 2972)	
Age (years)	58.0 ± 7.6	60.9 ± 6.9	<0.0001	57.5 ± 7.6	61.3 ± 7.0	<0.0001	57.6 ± 7.6	61.6 ± 7.1	<0.0001
Sex (female, %)	62.0	48.4	<0.0001	64.5	43.1	<0.0001	62.9	53.2	<0.0001
BMI (kg/m^2^)	25.7 ± 4.0	25.8 ± 3.6	0.13	25.6 ± 3.9	26.6 ± 4.1	<0.0001	25.6 ± 4.0	26.2 ± 3.9	<0.0001
Diet quality score	2.9 ± 1.4	2.9 ± 1.4	0.45	2.9 ± 1.4	2.9 ± 1.4	0.95	2.9 ± 1.4	2.9 ± 1.4	0.24
Total energy intake (kcal/day)	2275 ± 653	2347 ± 646	<0.01	2266 ± 648	2345 ± 679	<0.01	2272 ± 649	2309 ± 681	<0.0001
High physical activity (%)	52.5	53.3	0.74	52.8	51.1	0.08	52.4	53.8	0.15
Marital status (married, %)	65.2	70.4	0.02	65.4	64.8	0.48	65.3	65.8	0.58
Education level (%)									
Low	41.3	43.8	0.26	39.9	51.6	<0.0001	40.3	49.6	<0.0001
Median	44.2	43.0	0.61	44.8	39.4	<0.0001	44.7	39.9	<0.0001
High	14.5	13.2	0.41	15.3	9.03	<0.0001	15.0	10.4	<0.0001
Smoking status (%)									
Never smoker	28.4	15.8	<0.0001	27.4	33.9	<0.0001	28.0	29.9	0.03
Current smoker	33.3	35.6	0.28	33.2	34.3	0.21	33.6	30.9	<0.01
Former smoker	38.3	48.6	<0.0001	39.4	31.8	<0.0001	38.4	39.2	0.42
Never drinking alcohol (%)	6.22	5.76	0.68	5.99	7.69	<0.001	6.08	7.23	0.01
Diabetes (%)	4.04	5.56	0.09	3.32	9.25	<0.0001	3.61	7.67	<0.0001
Hypertension (%)	60.4	66.9	<0.01	58.2	76.7	<0.0001	58.5	76.7	<0.0001
Lipid-lowering drugs (%)	2.41	2.06	0.62	2.05	4.84	<0.0001	2.27	3.43	0.0001
NLR	2.1 ± 0.9	2.2 ± 0.9	0.02	2.1 ± 0.9	2.2 ± 1.0	<0.0001	2.1 ± 0.9	2.2 ± 1.0	0.08
ApoB/ApoA1 ratio	0.7 ± 0.2	0.7 ± 0.2	0.34	0.7 ± 0.2	0.8 ± 0.2	<0.0001	0.7 ± 0.2	0.7 ± 0.2	<0.0001
ApoA1 (mg/dL)	157.2 ± 28.1	156.7 ± 27.2	0.70	158.3 ± 28.1	149.3 ± 26.5	<0.0001	157.5 ± 28.1	154.6 ± 28.2	<0.0001
ApoB (mg/dL)	107.0 ± 26.1	108.4 ± 25.1	0.13	105.7 ± 25.9	116.2 ± 25.8	<0.0001	106.4 ± 26.1	111.5 ± 25.7	<0.0001
LDL cholesterol (mmol/L, *n* = 4,865)	4.2 ± 1.0	4.0 ± 0.9	0.11	4.1 ± 1.0	4.3 ± 0.9	<0.0001	4.2 ± 1.0	4.2 ± 1.1	0.09
HDL cholesterol (mmol/L, *n* = 4,934)	1.4 ± 0.4	1.3 ± 0.3	0.12	1.4 ± 0.4	1.3 ± 0.3	<0.0001	1.4 ± 0.4	1.3 ± 0.4	<0.0001
FBG (mmol/l, *n* = 4,981)	5.2 ± 1.3	5.4 ± 1.4	<0.001	5.1 ± 1.2	5.6 ± 2.0	<0.0001	5.1 ± 1.3	5.4 ± 1.7	<0.0001
Hemoglobin A1c (%, *n* = 4,981)	4.9 ± 0.7	4.9 ± 0.9	0.94	4.9 ± 0.7	5.1 ± 1.1	<0.0001	4.9 ± 0.7	5.0 ± 1.0	<0.0001

*BMI* body mass index, *FBG* fasting blood glucose, *HDL* high-density lipoprotein, *LDL* low-density lipoprotein, *NLR* neutrophil-to-lymphocyte ratio.

^a^Continuous variables are expressed as means (± standard deviations) and categorical variables are expressed as percentages.

^b^*P*-value was calculated using the Wilcoxon rank-sum test or logistic regression analysis where appropriate. The *P*-value has not been corrected for multiple testing.

### Incident PD, coronary events, and ischemic stroke

There were 486 incident PD cases (mean follow-up time: 21.1 ± 6.1 years), 3288 incident coronary events (mean follow-up time: 20.5 ± 6.6 years), and 2,972 incident ischemic stroke cases (mean follow-up time: 20.6 ± 6.5 years). The incidence rate per 10,000 person-years of follow-up was 8.8 cases for PD, 61.1 cases for coronary events, and 55.1 cases for ischemic stroke, respectively. In addition, among the 486 PD cases, 76 individuals developed coronary events and 77 individuals developed ischemic stroke.

### Risk factors for incident PD

**Fig. 1:**
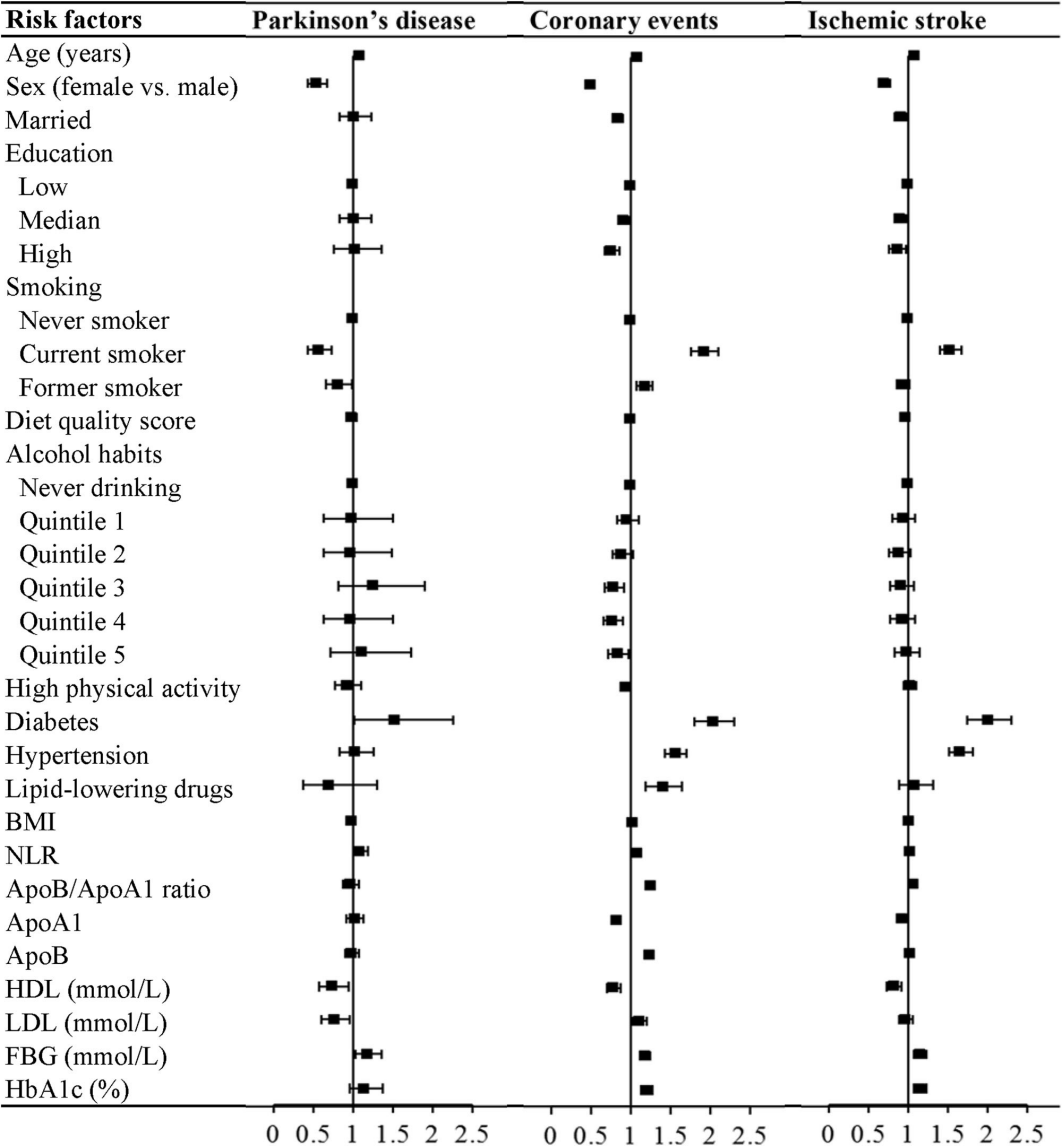
Forest plot for the associations between risk factors and outcomes (Parkinson’s disease, coronary events, and ischemic stroke). Multivariable Cox proportional hazards model included age, sex, marital status, education, smoking, diet quality score, alcohol habits, physical activity, diabetes, hypertension, lipid-lowering drugs, BMI, NLR, apoB/apoA1 ratio, and total energy intake. When analyzing ApoA1 and ApoB or HDL and LDL, ApoB/ApoA1 ratio was removed from the model. When analyzing FBG and HbA1c (separately), diabetes was removed from the model.

The multivariable analyses (Table 2 and Fig. 1) showed that age (in years) (hazard ratio [HR] = 1.08; 95% confidence interval [CI]: 1.07, 1.09), diabetes (HR = 1.52; 95% CI: 1.02, 2.26), neutrophil–lymphocyte ratio (NLR) (per standard deviation [SD] increase: HR = 1.09; 95% CI: 1.00, 1.19), and FBG (per SD increase: HR = 1.18; 95% CI: 1.03, 1.36) were associated with an increased risk of incident PD, whereas female sex (HR = 0.54; 95% CI: 0.43, 0.67), smoking (current smokers [HR = 0.57; 95% CI: 0.43, 0.74] and former smokers [HR = 0.81; 95% CI: 0.66, 0.99]), HDL (per SD increase: HR = 0.74; 95% CI: 0.57, 0.95), and LDL (per SD increase: HR = 0.77; 95% CI: 0.61, 0.96) were associated with an decreased risk of incident PD. In addition, married status, education, diet quality score, alcohol consumption, leisure-time physical activity, hypertension, dyslipidemia, BMI, ApoB/ApoA1 ratio, ApoA1, ApoB, and HbA1c factors were not significantly associated with risk of incident PD.

**Table 2:** Comparison of risk factors for Parkinson’s disease, coronary events, or ischemic stroke in the Malmö Diet and Cancer Study (*n* = 26,210).

Risk factors	Parkinson’s disease		Coronary events			Ischemic stroke		
	HR (95% CI)	*P*-value^a^	HR (95% CI)	*P*-value^a^	*P*-value for equal association^b^	HR (95% CI)	*P*-value^a^	*P*-value for equal association^b^
Age (years)	1.08 (1.06, 1.09)	<0.0001	1.08 (1.07, 1.08)	<0.0001	0.87	1.09 (1.09, 1.10)	<0.0001	0.06
Sex (female vs. male)	0.54 (0.43, 0.67)	<0.0001	0.50 (0.46, 0.55)	<0.0001	0.60	0.70 (0.64, 0.77)	<0.0001	0.02
Married	1.01 (0.83, 1.24)	0.90	0.84 (0.78, 0.90)	<0.0001	0.08	0.91 (0.84, 0.98)	0.01	0.30
Education					0.35			0.69
Low	1.00 (reference)	–	1.00 (reference)	–		1.00 (reference)	–	
Median	1.01 (0.83, 1.23)	0.93	0.91 (0.85, 0.98)	0.01		0.90 (0.84, 0.98)	0.01	
High	1.02 (0.76, 1.36)	0.91	0.76 (0.67, 0.86)	<0.0001		0.87 (0.76, 0.98)	0.03	
Smoking					<0.0001			<0.0001
Never smoker	1.00 (reference)	-	1.00 (reference)	–		1.00 (reference)	–	
Current smoker	0.57 (0.43, 0.74)	<0.0001	1.92 (1.76, 2.10)	<0.0001		1.53 (1.40, 1.68)	<0.0001	
Former smoker	0.81 (0.66, 0.99)	0.04	1.18 (1.08, 1.28)	<0.001		0.94 (0.86, 1.02)	0.15	
Diet quality score	0.98 (0.92, 1.05)	0.62	1.00 (0.97, 1.02)	0.68	0.75	0.97 (0.95, 1.00)	0.049	0.99
Alcohol intake					0.09			0.57
Never drinking	1.00 (reference)	–	1.00 (reference)	–		1.00 (reference)	–	
Quintile 1	0.98 (0.64, 1.50)	0.91	0.96 (0.83, 1.11)	0.61		0.94 (0.80, 1.09)	0.40	
Quintile 2	0.97 (0.64, 1.49)	0.90	0.89 (0.77, 1.03)	0.12		0.89 (0.76, 1.04)	0.15	
Quintile 3	1.25 (0.82, 1.90)	0.30	0.79 (0.68, 0.92)	<0.01		0.91 (0.77, 1.07)	0.24	
Quintile 4	0.97 (0.63, 1.50)	0.89	0.77 (0.66, 0.90)	<0.01		0.92 (0.78, 1.09)	0.34	
Quintile 5	1.11 (0.72, 1.74)	0.63	0.84 (0.72, 0.98)	0.02		0.98 (0.83, 1.15)	0.78	
High physical activity	0.92 (0.77, 1.11)	0.39	0.94 (0.88, 1.01)	0.08	0.85	1.02 (0.95, 1.10)	0.64	0.32
Diabetes	1.52 (1.02, 2.26)	0.04	2.04 (1.80, 2.31)	<0.0001	0.15	2.01 (1.75, 2.31)	<0.0001	0.18
Hypertension	1.03 (0.84, 1.26)	0.76	1.57 (1.44, 1.70)	<0.0001	<0.001	1.66 (1.52, 1.82)	<0.0001	<0.0001
Lipid-lowering drugs	0.70 (0.37, 1.31)	0.27	1.41 (1.19, 1.65)	<0.0001	0.02	1.09 (0.89, 1.32)	0.42	0.17
BMI (kg/m^2^)	0.99 (0.97, 1.02)	0.50	1.02 (1.01, 1.03)	<0.0001	0.03	1.01 (1.00, 1.02)	0.09	0.21
NLR^3^	1.09 (1.00, 1.19)	0.04	1.08 (1.04, 1.11)	<0.0001	0.78	1.03 (1.00, 1.07)	0.09	0.24
ApoB/ApoA1 ratio ^c^	0.97 (0.88, 1.07)	0.54	1.26 (1.23, 1.30)	<0.0001	<0.0001	1.07 (1.03, 1.11)	<0.001	0.07
ApoA1 ^c^	1.02 (0.92, 1.13)	0.69	0.83 (0.79, 0.86)	<0.0001	<0.0001	0.93 (0.89, 0.97)	<0.001	0.09
ApoB^3^	0.99 (0.90, 1.08)	0.76	1.24 (1.20, 1.28)	<0.0001	<0.0001	1.03 (0.99, 1.07)	0.11	0.38
HDL (mmol/L, *n* = 4865) ^3^	0.74 (0.57, 0.95)	0.02	0.78 (0.70, 0.87)	<0.0001	0.66	0.83 (0.74, 0.92)	<0.001	0.41
LDL (mmol/L, *n* = 4865)^c^	0.77 (0.61, 0.96)	0.02	1.11 (1.02, 1.20)	0.02	<0.01	0.97 (0.89, 1.06)	0.52	0.048
FBG (mmol/L, *n* = 4961)^c^	1.18 (1.03, 1.36)	0.02	1.18 (1.12, 1.25)	<0.0001	0.99	1.15 (1.08, 1.23)	<0.0001	0.74
HbA1c (%, *n* = 4961)^c^	1.14 (0.96, 1.37)	0.14	1.20 (1.14, 1.27)	<0.0001	0.60	1.16 (1.08, 1.24)	<0.0001	0.91

*BMI* body mass index, *CI* confidence interval, *FBG* fasting blood glucose, *HDL* high-density lipoprotein, *HbA1c* hemoglobin A1c, *HR* hazard ratio, *LDL* low-density lipoprotein, *NLR* neutrophil-to-lymphocyte ratio.

^a^Multivariable Cox proportional hazards model included age, sex, marital status, education, smoking, diet quality score, alcohol habits, physical activity, diabetes, hypertension, lipid-lowering drugs, body mass index, neutrophil-to-lymphocyte ratio, apoB/apoA1 ratio, and total energy intake. When analyzing ApoA1 and ApoB or HDL and LDL, ApoB/ApoA1 ratio was removed from the model. When analyzing fasting blood glucose and HbA1c (separately), diabetes was removed from the model.

^b^*P-*value associated with the null hypothesis that this variable has the same association with Parkinson’s disease, coronary events, or ischemic stroke with all other effects being different. Tests for education and smoking effects have 3 df, alcohol consumption has 6 df, and all others have 1 df. The *P*-value has not been corrected for multiple testing.

^c^Per one standard deviation increase.

### Comparing risk factors of incident PD and coronary events

With respect to the effects of the risk factors, the differences between PD and coronary events are shown in Table 2 and Fig. 1. Smoking and LDL were positively associated with risk of coronary events but were inversely associated with risk of PD (*P*-values for equal association < 0.01). Hypertension, lipid-lowering drugs, BMI, ApoB/ApoA1 ratio, and ApoB were positively associated with the risk of coronary events but were not associated with PD (*P-*values for equal association <0.05). In addition, ApoA1 was inversely associated with the risk of coronary events but was not associated with PD (*P*-value for equal association <0.0001). By contrast, age, male sex, diabetes, NLR, and FBG were common risk factors for coronary events and PD (*P-*values for equal association ≥0.15).

### Comparing risk factors of incident PD and ischemic stroke

The comparisons of risk factors for PD and ischemic stroke are presented in Table 2 and Fig. 1. Female sex was more protective for PD (HR = 0.54; 95% CI: 0.43, 0.67) when compared with ischemic stroke (HR = 0.70; 95% CI: 0.64, 0.77) (*P*-value for equal association, 0.02). Smoking was positively associated with risk of ischemic stroke but was inversely associated with risk of PD (*P-*value for equal association <0.0001). Age, diabetes, NLR, and FBG were the shared risk factors in PD and ischemic stroke (*P-*values for equal association ≥0.06), whereas HDL was a common protective factor for the two outcomes (*P*-value for equal association = 0.41). In addition, hypertension was positively associated with the risk of ischemic stroke but was not significantly associated with the risk of PD (*P-*value for equal association < 0.0001). In contrast, LDL was inversely associated with the risk of PD but was not significantly associated with the risk of ischemic stroke (*P*-value for equal association = 0.048).

In sensitivity analyses, similar results were observed when excluding those who developed both PD and coronary events or ischemic stroke during the follow-up (data not shown).

## Discussion

In this large prospective cohort study with a mean follow-up of 21 years, we found that age, diabetes, NLR, and FBG were associated with a higher risk of PD, whereas female sex, smoking, HDL, and LDL were associated with a lower risk of PD. For the comparison of risk factors, we found that smoking, hypertension, and LDL were differently associated with PD versus both coronary events and ischemic stroke. In addition, the associations with female sex, lipid-lowering drugs, BMI, ApoB/ApoA1 ratio, ApoA1, and ApoB were different for PD versus coronary events and PD versus ischemic stroke. To our knowledge, this is the first study investigating the association of traditional CVD risk factors, inflammatory biomarkers, and lipid profiles with incident PD in a large prospective population-based cohort, and comparing the predictive strength of risk factors for the incidence of two disease pairs, PD versus coronary events and PD versus ischemic stroke, respectively.

In line with several previous studies, our results showed that age^3^, diabetes^10^, and FBG^11,12^ were risk factors for PD, whereas female sex was a protective factor for PD^13^. For competing risk analysis, age, diabetes, and FBG had similar associations with PD, coronary events and ischemic stroke. The female sex had a stronger association with PD as compared to ischemic stroke. Individuals with diabetes are older and have disrupted insulin signaling, which is related to the pathophysiology of PD. A high FBG level indicates a relatively high risk for the future development of diabetes^14^. Thus, our findings highlighted that people with a high FBG level had a high possibility of developing PD. Furthermore, we did not observe an association between HbA1c and PD. This was supported by the fact that FBG and HbA1c represent different pathophysiological aspects of glycemic control^14,15^.

The involvement of chronic low-grade systemic inflammation in the development of PD has gained a lot of interest. NLR is a valid, non-invasive marker of peripheral Inflammation^16^. It has been reported that NLR was significantly higher in PD patients compared to healthy controls^17,18^. A recent study also showed that components of peripheral blood leukocytes reflect some clinical symptoms of PD^19^. Moreover, the neuroimaging studies showed that NLR was correlated with loss of dopaminergic uptake and connectivity of white matter tracts in certain brain regions in PD patients, indicating the involvement of peripheral inflammation in the development of neurodegeneration^20,21^. However, the predictive value of NLR for PD risk has not been prospectively studied before. Our study is the first that found higher NLR level was associated with the risk of incident PD in the general population. NLR was associated with the risk of coronary events or ischemic stroke. Our findings suggest that chronic inflammation is the common underlying pathophysiologic mechanism for PD and CVD.

We observed that smoking was protective against the risk of PD, consistent with previous studies^22,23^. However, it was difficult to make a causal conclusion given the possibility of reverse causation and confounding effects. The evidence that parental smoking was inversely related to the incidence of PD in the offspring seems to argue against a major role of reverse causation^24^. Moreover, a recent study reported a dose-response relationship between PD risk and smoking duration and intensity, suggesting a causal link between smoking and PD^25^. For the competing risk comparison, smoking had opposite associations with PD versus coronary events and ischemic stroke, which was in line with previous evidence that smoking is a well-known modified risk factor for incident CVD^26^. However, it is inappropriate to recommend smoking for the prevention of PD, given the deleterious CVD effect of smoking. Therefore, future studies are warranted to clarify the biological mechanism responsible for the protective effect.

Our results showed that LDL was inversely associated with the risk of PD, which was in accordance with previous studies^27,28^. Moreover, a recent Mendelian randomization study suggested that a higher LDL level might be indeed a causal pathway to PD^29^. In addition, we observed an inverse association between HDL and PD risk. However, most previous studies indicated a lack of association between HDL and PD risk^27–29^. In contrast, three prospective studies reported that low HDL was associated with an increased risk of PD^11,12,30^, which supports our current findings. Contrary to HDL and LDL, we did not find any association between ApoA1, ApoB, ApoB/ApoA1 ratio, and risk of PD in the current study. Previous case-control studies showed that ApoA1 level was lower in PD patients than in normal individuals, and lower ApoA1 level was associated with earlier age at PD onset^31,32^. However, prospective cohort studies including ours and others did not find any association between ApoA1 and incident PD^29^. Furthermore, one prospective study found ApoB was related to a lower risk of PD, but did not find causality in the Mendelian randomization analysis^29^. Another case-control study showed that PD patients had a lower ApoB level^33^. Moreover, in line with our current study, a prospective cohort study found no association between ApoB/ApoA1 ratio and risk of PD^29^. For the analysis of competing risk factors for PD versus coronary events or ischemic stroke, LDL was differentially related to the three outcomes. In addition, we observed that ApoB, ApoA1, and their ratio had a significant difference for PD-coronary events but not PD-ischemic stroke. Our findings were supported by the evidence that ApoB and ApoB/ApoA1 ratio are risk factors for CVD, while ApoA1 is a protective factor for CVD^34^.

We also investigated the PD risk in relation to other CVD risk factors, such as hypertension, diet quality, alcohol consumption, and leisure-time physical activity. Regarding the association of hypertension with the risk of PD, most of the previous studies also reported a null association^6,35^. However, hypertension is one of the most important risk factors for CVD. Contrary to previous studies^36,37^, we did not find a significant association between diet quality and risk of PD. Of note, most of the studies where high diet quality was associated with a lower risk of PD were case-control studies or had less than 10 years of follow-up^37^, which is shorter than the follow-up in our study (mean: 21 years). Since PD has a long prodromal phase, it is important that the follow-up is not too short to prevent reverse causation. Furthermore, we observed no association between alcohol consumption and PD risk. Similarly, two prospective cohort studies with a mean follow‐up of >10 years also did not find an association between alcohol intake and PD risk^38,39^. In contrast, a recent Korean prospective study with a 14-year follow-up reported that alcohol consumption showed an inverse association with PD^40^, which is supported by meta-analysis results of case-control studies^41^. The results should be confirmed in other studies before any recommendations can be made. Moreover, consistent with our study, a Sweden prospective cohort study showed that leisure-time exercise was not associated with PD risk^42^.

The strength of the study includes the prospective design, a long follow-up period, and the large population-based cohort with a reasonable number of endpoints retrieved from high-quality registers. Further, the inclusion of the modified Lunn-McNeil method in the analysis provides a reliable tool for comparison of risk factors for PD, coronary events, and ischemic stroke within the same population to give a quantitative estimate of the strength of association.

Nevertheless, the study has several limitations. First, lifestyle factors were measured at baseline and did not account for possible changes during follow-up. However, the use of baseline information may help avoid reverse causation resulting from changes in lifestyle factors after disease onset. In addition, we examined associations of blood indicators measured at a single timepoint (only once at baseline) with outcome risk, which are weaker than associations for repeatedly measured indicators because of regression dilution bias^43^. Future studies should be conducted with repeated measurements of the blood level. Second, as with any observational study, we cannot conclude causality and cannot rule out residual confounding. Third, our study population consists primarily of European descent, which might limit the generalizability of our findings. Fourth, the identification of PD cases was via electronic record linkage rather than an in-person screening (clinical diagnoses), which might overestimate the PD cases^44^. However, this method has the advantage of allowing analysis on everyone recruited to the study. Moreover, previous Swedish studies showed an acceptable sensitivity and specificity for registers sources of PD as compared with clinical diagnoses^45^. Nevertheless, information on the clinical diagnosis (whether it is PD or PD plus syndrome) was not available in this study. Fifth, although we did not include PD cases with ICD-9 code 332.1 and ICD-10 code G21 to exclude vascular PD, other defined secondary Parkinsonism, and unspecified secondary Parkinsonism, it is impossible to ascertain that vascular PD cases were completely ruled out. Finally, data on the severity of smoking (average number of cigarettes per day) was not available in the current study. Therefore, it was possible that lesser smokers may feel protective effect and even improvement of PD, while heavy smokers may suffer from increased CVD risk.

In conclusion, age, diabetes, NLR, and FBG were positively associated with risk of PD, whereas female sex, smoking, HDL, and LDL were inversely associated with risk of PD. In addition, our results showed the risk factor profiles for PD, coronary events, and ischemic stroke had many similarities, but also important differences. Future studies should explore the causality and mechanism behind the relationship to finely balance these risk factors in the elderly population.

## Methods

### Study population

The Malmö Diet and Cancer Study (MDCS) is a population-based prospective cohort study conducted in the city of Malmö, Sweden. Between 1991 and 1996, all men born 1923–1945 and women born 1923–1950 living in Malmö were invited to participate in the study. Approximately 40% of the source population participated in the study. At baseline visits (1991–1996), participants answered a comprehensive questionnaire and underwent health examinations. All procedures performed in this study were approved by the regional ethics committee in Lund, Sweden (LU 51/90) and carried out in accordance with the Helsinki Declaration. All participants provided written informed consent to take part in the study.

**Fig. 2:**
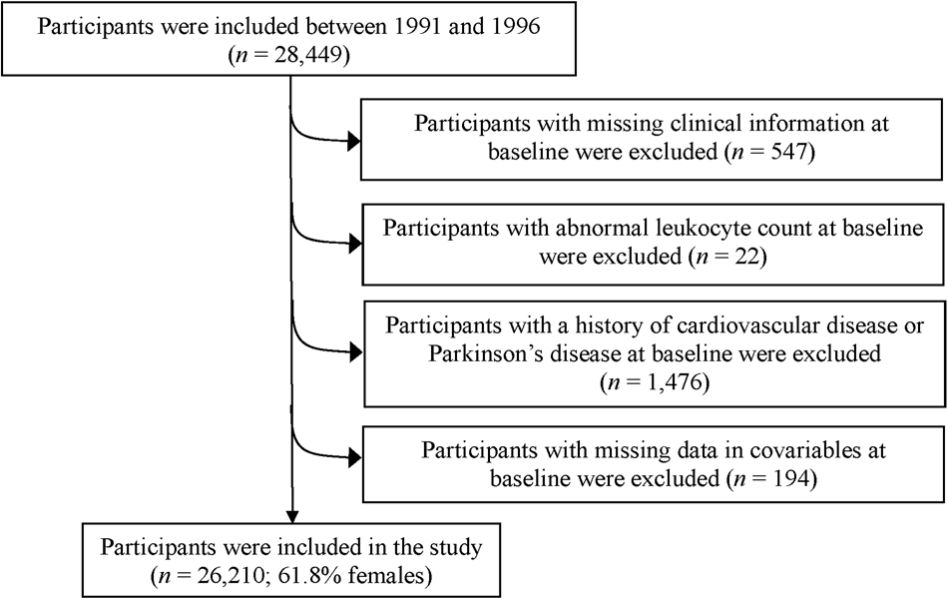
The flow chart of study participants.

For the current study, out of the initial 28,449 participants (11,246 males and 17,203 females), 547 individuals were excluded for lack of clinical information and leukocyte count data, and 22 individuals were excluded for the abnormality of total leukocyte count (>20 × 10^9^/L) to rule out acute inflammation or probable laboratory errors^46^. We further excluded 1,476 participants with a history of prevalent CVD or PD at baseline and 194 participants with missing covariate data. The final study population consisted of 26,210 participants (10,019 males and 16,191 females, aged 45–73 years). Figure 2 displays the sampling procedure for the present study.

### Baseline examinations

Age and sex were collected by procuring the civic registration numbers of each participant. Data on married status, education level, lifestyle factors, medical history, and medication usage were obtained from a self-administered questionnaire. Smoking status was recorded as current smokers, former smokers, and never smokers. Alcohol consumption was divided into 6 groups (zero-consumers and sex-specific quintiles of consumers [quintile 1: <0.9 g/day for women or <3.4 g/day for men; quintile 2: 0.9–4.3 g/day for women or 3.4–9.1 g/day for men; quintile 3: 4.3–8.1 g/day for women or 9.1–15.7 g/day for men; quintile 4: 8.1–14.0 g/day for women or 15.7–25.7 g/day for men; quintile 5: >14.0 g/day for women or >25.7 g/day for men]). History of prevalent diabetes was accessed through self-reported physician diagnosis, defined as receiving antidiabetic medication currently, or a fasting whole blood glucose level ≥6.1 mmol/L (corresponding to plasma glucose ≥7.0 mmol/L). Blood pressure was measured after 10 min-rest in the supine position. Hypertension was defined as systolic blood pressure ≥140 mmHg and/or diastolic blood pressure ≥90 mmHg and/or taking antihypertensive medications. Body weight and height were measured by trained nurses according to standard procedures. BMI was calculated as weight (kg) divided by height (cm) squared. Leisure-time physical activity was assessed using questionnaire items adapted from the Minnesota Leisure Time Physical Activity Questionnaire, and calculated as the weekly metabolic equivalent hours (MET-hour/week). A high leisure-time physical activity was defined as ≥25 MET-hour/week. Diet quality was assessed using a Swedish diet quality score, which was developed and validated for the MDCS cohort.

### Laboratory measurements

Baseline non-fasting blood samples were collected from the MDCS participants. On the same day of blood sampling, blood cell counts were measured with the standard protocol. SYSMEX K1000 automated hematology analyzer (Sysmex Europe, Norderstedt, Germany) was used to quantify the total and each subtype of leukocyte count in heparinized blood samples. The coefficient of variations for 20 consecutive counts of total leukocytes, neutrophils, lymphocytes, and mixed cells in one blood sample were 1.45, 1.85, 6.22, and 16.1%, respectively. The measurement ranged from 1.00 × 10^9^ to 99.9 × 10^9^ /L for total leukocyte count. NLR was calculated as the ratio of neutrophil and lymphocyte counts measured from the same blood sample. Frozen plasma samples, stored at minus 80 °C immediately after collection, were used to measure ApoA1 and ApoB concentrations. The inter-assay coefficients of variability were <4.0% for both ApoA1 and ApoB. The ApoB/ApoA1 ratio was calculated as the ratio of ApoB/ApoA1 measured in the same blood sample. LDL cholesterol, HDL cholesterol, FBG, and HbA1c were determined from fasting blood samples in the Malmö Diet and Cancer Cardiovascular cohort study, a sub-cohort of the MDCS.

### Follow-up and ascertainment of outcomes

Participants were followed up from baseline examination until death, migration from Sweden, the first diagnosis of PD, coronary events or ischemic stroke, or end of follow-up (December 31st, 2018). The local and national registers including the Swedish National Hospital Discharge Register, the Swedish National Cause of Death Register, and the Stroke Register of Malmö were used to retrieve the information on incident PD, coronary events, and ischemic stroke cases throughout the follow-up period^47,48^. PD was defined based on the International Classification of Diseases 9th (ICD-9) code 332.0 and ICD-10 G20. Coronary events were defined as ICD-9 codes 410A-410X and ICD-10 code I21 or death attributable to ischemic heart disease (ICD-9 codes: 410–414; ICD-10 codes: I20-I25). Ischemic stroke was defined as ICD-9 code 434 and ICD-10 code I63.

### Statistical analysis

Baseline characteristics were reported using means ± SDs for continuous variables and percentages for categorical variables. The normality of continuous variables was assessed using the Kolmogorov–Smirnov test (*n* ≥ 2,000). Differences in the distribution of baseline characteristics between participants with and without incident PD, coronary events, or ischemic stroke were compared using the Wilcoxon rank-sum test for continuous variables due to their skewed distributions or logistic regression analysis for categorical variables.

Multivariable Cox proportional hazard regression models were used to calculate HRs with 95% CIs for incident PD, coronary events, or ischemic stroke. The proportional hazards assumption was checked using the Schoenfeld residuals^49^, and no violation was found. The multivariable model included age, sex, marital status, education level, smoking status, diet quality score, alcohol consumption, leisure-time physical activity, diabetes, hypertension, lipid-lowering drugs, BMI, NLR, ApoB/ApoA1 ratio, and total energy intake at baseline examination. To avoid bias due to over-adjustment, when analyzing ApoA1 and ApoB or HDL and LDL, the ApoB/ApoA1 ratio was removed from the model; when analyzing FBG and HbA1c (separately), diabetes was removed from the model. All these variables were simultaneously entered into the multivariable model. Therefore, when calculating the estimate of any one variable, the other variables entered into the multivariable model were the controlled variables.

We further examined whether the risk associated with each covariate was similar for two pairs (PD-coronary events and PD-ischemic stroke). For this purpose, we used a modified method of Lunn-McNeil competing risks models using a data duplication method^50,51^. In brief, this consists of duplicating the dataset, so that each individual appears in two strata. The failures (PD-coronary events or PD-ischemic stroke) were then sorted by strata, and a stratified Cox regression was performed, which thus allows the estimation of separate HRs for the two sets of outcomes. Finally, the likelihood ratio test was used to compare this model which allows the association with the exposures of interest to vary according to the outcome with a model that does not. *P* values for the difference in the effect of a given exposure to the separate outcomes are derived from this likelihood ratio test, and the null hypothesis is that both outcomes are equally associated with the risk factors. Compared to the original Lunn-McNeill model^50^, the modified version has events in both strata if the participants had developed both PD-coronary events or PD-ischemic stroke. The HRs obtained from this approach are identical to results from separate Cox regression models run for each outcome. Furthermore, to test the robustness of our results, we performed a sensitivity analysis by excluding those with both incident PD and incident coronary events or ischemic stroke during follow-up.

All statistical analyses were performed using SAS software, version 9.4 (SAS Institute Inc., Cary, NC, USA). A two-tailed *P* < 0.05 was recognized as statistically significant.

## Data Availability

The data that support the findings of this study are available from “The Malmö Cohorts” at Lund University with the permission of the MDC Steering Committee (https://www.malmo-kohorter.lu.se/malmo-cohorts).

## References

[1] Collaborators GBDPsD. (2018). Global, regional, and national burden of Parkinson’s disease, 1990–2016: a systematic analysis for the Global Burden of Disease Study 2016. Lancet Neurol..

[2] Macerollo A, Chen JC (2016). Trends in the incidence of Parkinson disease. JAMA Neurol..

[3] Potashkin J (2020). Understanding the links between cardiovascular disease and Parkinson’s disease. Mov. Disord..

[4] Park JH (2020). Association of Parkinson disease with risk of cardiovascular disease and all-cause mortality: a nationwide, population-based cohort study. Circulation.

[5] Liu Y, Xue L, Zhang Y, Xie A (2020). Association between stroke and Parkinson’s disease: a meta-analysis. J. Mol. Neurosci..

[6] Kizza J (2019). Cardiovascular risk factors and Parkinson’s disease in 500,000 Chinese adults. Ann. Clin. Transl. Neurol..

[7] Muller J, Myers J (2018). Association between physical fitness, cardiovascular risk factors, and Parkinson’s disease. Eur. J. Prev. Cardiol..

[8] Vikdahl M, Backman L, Johansson I, Forsgren L, Haglin L (2015). Cardiovascular risk factors and the risk of Parkinson’s disease. Eur. J. Clin. Nutr..

[9] Marko-Kucsera M, Vecsei L, Paulik E (2018). Association of cardiovascular risk factors and Parkinson’s disease - case-control study in South East Hungary. Ideggyogy. Sz..

[10] Chohan H (2021). Type 2 diabetes as a determinant of Parkinson’s disease risk and progression. Mov. Disord..

[11] Nam GE (2018). Metabolic syndrome and risk of Parkinson disease: a nationwide cohort study. PLoS Med..

[12] Roh JH, Lee S, Yoon JH (2021). Metabolic syndrome and Parkinson’s disease incidence: a nationwide study using propensity score matching. Metab. Syndr. Relat. Disord..

[13] Hirsch L, Jette N, Frolkis A, Steeves T, Pringsheim T (2016). The incidence of Parkinson’s disease: a systematic review and meta-analysis. Neuroepidemiology.

[14] American Diabetes A. (2014). Diagnosis and classification of diabetes mellitus. Diabetes Care.

[15] Association American D. (2018). Updates to the standards of medical care in diabetes-2018. Diabetes Care.

[16] Alkhouri N (2012). Neutrophil to lymphocyte ratio: a new marker for predicting steatohepatitis and fibrosis in patients with nonalcoholic fatty liver disease. Liver Int..

[17] Akil E (2015). The increase of carcinoembryonic antigen (CEA), high-sensitivity C-reactive protein, and neutrophil/lymphocyte ratio in Parkinson’s disease. Neurol. Sci..

[18] Munoz-Delgado L (2021). Peripheral immune profile and neutrophil-to-lymphocyte ratio in Parkinson’s disease. Mov. Disord..

[19] Umehara T, Oka H, Nakahara A, Matsuno H, Murakami H (2020). Differential leukocyte count is associated with clinical phenotype in Parkinson’s disease. J. Neurol. Sci..

[20] Sanjari Moghaddam H, Ghazi Sherbaf F, Mojtahed Zadeh M, Ashraf-Ganjouei A, Aarabi MH (2018). Association between peripheral inflammation and DATSCAN data of the striatal nuclei in different motor subtypes of Parkinson disease. Front Neurol..

[21] Haghshomar M (2019). White matter changes correlates of peripheral neuroinflammation in patients with Parkinson’s disease. Neuroscience.

[22] Li X, Li W, Liu G, Shen X, Tang Y (2015). Association between cigarette smoking and Parkinson’s disease: a meta-analysis. Arch. Gerontol. Geriatr..

[23] Checkoway H (2002). Parkinson’s disease risks associated with cigarette smoking, alcohol consumption, and caffeine intake. Am. J. Epidemiol..

[24] O’Reilly EJ (2009). Smoking and Parkinson’s disease: using parental smoking as a proxy to explore causality. Am. J. Epidemiol..

[25] Gallo V (2019). Exploring causality of the association between smoking and Parkinson’s disease. Int J. Epidemiol..

[26] Collaborators GBDT. (2017). Smoking prevalence and attributable disease burden in 195 countries and territories, 1990–2015: a systematic analysis from the Global Burden of Disease Study 2015. Lancet.

[27] Jiang Z (2020). Effects of higher serum lipid levels on the risk of Parkinson’s disease: a systematic review and meta-analysis. Front Neurol..

[28] Fu X (2020). A systematic review and meta-analysis of serum cholesterol and triglyceride levels in patients with Parkinson’s disease. Lipids Health Dis..

[29] Fang F (2019). Lipids, apolipoproteins, and the risk of Parkinson disease. Circ. Res.

[30] Park JH (2021). Association of high-density lipoprotein cholesterol variability and the risk of developing Parkinson disease. Neurology.

[31] Swanson CR (2015). Lower plasma apolipoprotein A1 levels are found in Parkinson’s disease and associate with apolipoprotein A1 genotype. Mov. Disord..

[32] Swanson CR (2015). Plasma apolipoprotein A1 associates with age at onset and motor severity in early Parkinson’s disease patients. Mov. Disord..

[33] Li J (2020). Correlations between blood lipid, serum cystatin C, and homocysteine levels in patients with Parkinson’s disease. Psychogeriatrics.

[34] Walldius G (2012). The apoB/apoA-I ratio is a strong predictor of cardiovascular risk. J Intern. Med.

[35] Simon KC, Chen H, Schwarzschild M, Ascherio A (2007). Hypertension, hypercholesterolemia, diabetes, and risk of Parkinson disease. Neurology.

[36] Strikwerda AJ, Dommershuijsen LJ, Ikram MK, Voortman T (2021). Diet quality and risk of Parkinson’s disease: the Rotterdam study. Nutrients.

[37] Liu YH (2021). Diet quality and risk of Parkinson’s disease: a prospective study and meta-analysis. J. Parkinsons Dis..

[38] Kim IY (2020). Alcohol intake and Parkinson’s disease risk in the million women study. Mov. Disord..

[39] Peters S (2020). Alcohol consumption and risk of parkinson’s disease: data from a large prospective European cohort. Mov. Disord..

[40] Yoon SY, Park YH, Lee HJ, Kang DR, Kim YW (2021). Lifestyle factors and Parkinson disease risk: Korean Nationwide Cohort Study with repeated health screening data. Neurology.

[41] Jimenez-Jimenez FJ, Alonso-Navarro H, Garcia-Martin E, Agundez JAG (2019). Alcohol consumption and risk for Parkinson’s disease: a systematic review and meta-analysis. J. Neurol..

[42] Yang F (2015). Physical activity and risk of Parkinson’s disease in the Swedish National March Cohort. Brain.

[43] Prospective Studies C (2007). Blood cholesterol and vascular mortality by age, sex, and blood pressure: a meta-analysis of individual data from 61 prospective studies with 55,000 vascular deaths. Lancet.

[44] Gallo V (2015). Parkinson’s disease case ascertainment in the EPIC Cohort: the NeuroEPIC4PD study. Neurodegener. Dis..

[45] Feldman AL (2012). Accuracy and sensitivity of Parkinsonian disorder diagnoses in two Swedish national health registers. Neuroepidemiology.

[46] Zia E, Melander O, Bjorkbacka H, Hedblad B, Engstrom G (2012). Total and differential leucocyte counts in relation to incidence of stroke subtypes and mortality: a prospective cohort study. J. Intern Med.

[47] Ludvigsson JF (2011). External review and validation of the Swedish national inpatient register. BMC Public Health.

[48] Jerntorp P, Berglund G (1992). Stroke registry in Malmo, Sweden. Stroke.

[49] Hess KR (1995). Graphical methods for assessing violations of the proportional hazards assumption in Cox regression. Stat. Med.

[50] Lunn M, McNeil D (1995). Applying Cox regression to competing risks. Biometrics.

[51] Glynn RJ, Rosner B (2005). Comparison of risk factors for the competing risks of coronary heart disease, stroke, and venous thromboembolism. Am. J. Epidemiol..

